# Functional Characterization of Novel Chitinase Genes Present in the Sheath Blight Resistance QTL: qSBR11-1 in Rice Line Tetep

**DOI:** 10.3389/fpls.2016.00244

**Published:** 2016-03-01

**Authors:** Kamboj Richa, Ila M. Tiwari, Mandeep Kumari, B. N. Devanna, Humira Sonah, Archana Kumari, Ramawatar Nagar, Vinay Sharma, Jose R. Botella, Tilak R. Sharma

**Affiliations:** ^1^ICAR-National Research Centre on Plant BiotechnologyNew Delhi, India; ^2^Department of Bioscience and BiotechnologyBanasthali Vidyapith, Vanasthali, India; ^3^School of Agriculture and Food Sciences, The University of QueenslandBrisbane, QLD, Australia

**Keywords:** chitinase, *Rhizoctonia solani*, sheath blight, Tetep, xylanase

## Abstract

Rice sheath blight disease caused by *Rhizoctonia solani* is one of the most devastating diseases in rice leading to heavy yield losses. Due to the polygenic nature of resistance, no major resistance gene with complete host resistance against *R. solani* has been reported. In this study, we have performed molecular and functional analysis of the genes associated with the major *R. solani*-resistance QTL qSBR11-1 in the indica rice line Tetep. Sequence analysis revealed the presence of a set of 11 tandem repeats containing genes with a high degree of homology to class III chitinase defense response genes. Real-time quantitative PCR analysis showed that all the genes are strongly induced 36 h after *R. solani* infection. Comparison between the resistant Tetep and the susceptible HP2216 lines shows that the induction of the chitinase genes is much higher in the Tetep line. Recombinant protein produced *in vitro* for six of the eleven genes showed chitinolytic activity in gel assays but we did not detect any xylanase inhibitory activity. All the six *in vitro* expressed proteins show antifungal activity with a clear inhibitory effect on the growth of the *R. solani* mycelium. The characterized chitinase genes can provide an important resource for the genetic improvement of *R. solani* susceptible rice lines for sheath blight resistance breeding.

## Introduction

Rice is one of the important food crops and provides an essential part of the daily dietary intake for nearly half of the world's population (Maclean et al., 2002). However, rice production worldwide is affected by various biotic and abiotic stresses. Rice sheath blight disease, caused by *Rhizoctonia solani*, is one of the most destructive diseases affecting rice, leading to large scale yield losses especially in the areas such as the U.S.A, Japan, China, and India, where intensive agricultural practices are being followed (Gautam et al., 2003). Under favorable environmental conditions, sheath blight fungus can reduce yield by up to 50% (Lee and Rush, 1983; Marchetti and Bollich, 1991). *R. solani* is a soil borne necrotrophic pathogen and it survives either as sclerotia or mycelia in the debris of host plants. The sclerotia float to the surface of flood water in the rice fields and germinate on rice sheaths forming infection cushions or appressoria during the infection process. After the initial infection, the pathogen colonizes the entire plant through surface hyphae, developing new infection structures and significant necrotic damage (Ou, 1985). *Rhizoctonia* is a species complex and is able to infect at least 27 plant families, including many economically important monocots and eudicots. Due to this wide host range, high genetic variability, long survival ability in the form of sclerotia and the low inherent resistance level of rice cultivars against this disease, management of sheath blight is quite inefficient (Taheri et al., 2007).

Qualitative resistance to *R. solani* is not available in the rice germplasm available worldwide (Pan et al., 1999). However, quantitative resistance to *R. solani* has been reported in some rice varieties (Kumar et al., 2003) and many QTLs have been found to confer partial resistance in different rice cultivars, including Tetep, Teqing, jasmine, etc. (Li et al., 1995; Zou et al., 2000; Che et al., 2003; Pinson et al., 2005; Xie et al., 2008; Liu et al., 2009; Yin et al., 2009; Channamallikarjuna et al., 2010; Wang et al., 2012; Yadav et al., 2015). We have previously mapped a major QTL (qSBR11-1) providing substantial resistance to sheath blight; to chromosome 11 of the indica rice line Tetep and it is flanked by markers K39516 and sbq33 (~0.85 Mb; Channamallikarjuna et al., 2010). The genomic region associated with qSBR11-1 contains 11 tandem repeats with open reading frames encoding for proteins structurally similar to the plant class III chitinases (Channamallikarjuna et al., 2010).

In the present study we report the cloning and functional characterization of the 11 tandem repeat genes present in the QTL qSBR11-1 and analysis of the biochemical and antifungal activities of their encoded proteins.

## Materials and methods

### Plant material

Seeds of indica rice line Tetep and HP2216 were available with the authors at National Research Centre on Plant Biotechnology (NRCPB), New Delhi. Rice sheath blight pathogen *R. solani* strain (Rs-K) was collected from Rice Research Station Kapurthala, Punjab Agricultural University, Ludhiana, India.

### Rice plant inoculation and sample collection

Sclerotial inoculum of *R. solani* was prepared from fungal culture grown on oatmeal agar medium. This inoculum was used to inoculate 60 days old resistant indica rice cv. Tetep and susceptible rice cv. HP2216 plants under field conditions. For inoculation, uniform sized sclerotial plugs were placed beneath the leaf sheaths of at least three randomly selected tillers for each treatment. As a control, an agar plug without sclerotia was also placed in the sheath region. For each set of experiment, uninoculated control plants were also grown and maintained under similar conditions. Leaf samples from all the experiments was collected at 0, 12, 24, 36, and 48 h post inoculation (hpi) and stored at −80°C for further use.

### Quantitative RT–PCR

Total RNA was isolated from the control and inoculated leaves using RNeasy Plant Mini Kit (Qiagen). Quality and quantity of RNA was analyzed by agarose gel (1.0%) electrophoresis and nanodrop (Thermo Scientific), respectively. cDNA was prepared from 1 μg of each of total RNA sample using protoscript first strand cDNA synthesis kit (New England Biolabs, UK). The relative expression of 11 candidate chitinase genes present in QTL qSBR11-1 was analyzed by qRT-PCR at 0, 12, 24, 36, and 48 hpi. 18srRNA primer was used as internal control to normalize the data. Specific primers were designed for each gene (Supplementary Table 2). cDNA prepared was used as a template for expression analysis by Light cycler® SYBER green I master Kit using Light Cycler 480 II PCR system (Roche) using manufacturer's guidelines. The qRT-PCR cycling conditions were: initial denaturation 95°C; 5 min), 50 cycles of 95°C; 10 s, and annealing temperature (Supplementary Table 2) 15 s, 72°C 15 s.

### Cloning of defense response genes

Based on the qRT-PCR data, six DR genes were selected for cloning and characteization. These six genes were amplified and cloned in to bacterial expression vector pET29a using CDS specific primer having restriction site for *EcoR*I and *Hind*III restriction enzyme in forward and reverse primers, respectively (Supplementary Table 3) using cDNA of rice line Tetep as a template and transformed into *Escherichia coli* (strain DH5α). Plasmid DNA was isolated from transformed colonies using alkaline lysis method (Sambrook and Russell, 2001) and cloning was confirmed by restriction digestion and also by PCR analysis.

### Computational analysis of the genes

Full length nucleotide sequence of 11 defense response genes was retrieved from the rice genome database (www.gramene.org). FGENESH gene prediction software (www.softberry.com) was used to predict the candidate gene structures. For *in silico* expression studies, BLAST analysis was performed for all the 11 defense response genes using the full length cDNA sequences as well as ESTs against rice genome database available in NCBI (www.ncbi.nlm.nih.gov). Conserved domain analysis was also performed to identify the common structural domains found in defense response genes (www.ncbi.nlm.nih.gov/cdd). Further, we performed promoter analysis using 2 kb DNA sequences upstream to the Translational Start Site (TSS) of all 11 genes of indica rice which was retrieved from rice genome database (www.gramene.org) and analysis was performed using PlantCARE database (http://bioinformatics.psb.ugent.be/webtools/plantcare/html/).

### *In vitro* expression analysis of defense response genes

The recombinant plasmid with independent Defense Response (DR) gene of interest as well as native pET29a (control) were further transformed into *E. coli* strain BL21 DE3.0 for *in vitro* expression analysis. *E. coli* cells grown in LB medium containing 50 μg/ml of kanamycin (OD_600_ −0.6) were induced with IPTG (0.1 mM), cultured at 37°C and 180 rpm for 4 h and harvested by centrifuging at 11,000 × g for 10 min at 4°C. The pellet was resuspended in 1X PBS and 4X SDS sample buffer for denaturation and incubated at 95°C for 10–15 min and used for crude protein extraction. Induced and un-induced protein samples were separated on the 12% SDS-PAGE and subsequently staining and destaining was done as per the standard protocol (Sambrook and Russell, 2001).

### Western blotting

Total crude protein extract was separated by 12% SDS-PAGE and transferred on to a nitrocellulose membrane using electro-blotting apparatus (Bio-Rad). Membrane was blocked for 16 h at room temperature using 1X PBST buffer [1XPBS and 0.1% (v/v) Tween 20] and with 5% skim milk and washed three times with 1X PBST for 15 min each. Membrane was then incubated with anti-his tag specific primary antibody conjugated (1:5000 dilution) with horseradish peroxidase enzyme (Invitrogen, USA) for 2 h. The membrane was again washed three times with 1X PBST for 15 min each. Blot was then developed with HRP substrate (Invitrogen, USA) for 2–3 min under dark condition.

### Xylanase inhibition assay

LB agar (1.5%) plate containing 1% (w/v) birchwood xylan substrate was used to perform xylanase inhibition assay. In separate treatments, 10 μM of xylanase enzyme from *Thermomyces langiginosus* (Sigma) alone (control), xylanase coupled separately with 20 μg crude protein extract of the six defense response genes, xylanase inhibitor (15 mM N-bromosuccinimide, Sigma) as a positive control and with 5 μg BSA were incubated in 1X PBS buffer (pH 8.0) for 1 h at 30°C. These samples were then transferred into the wells punched on the LB agar—xylan plate and incubated in the dark for 24 h at 30°C. After the incubation plate was stained with 1% Congo red solution for 15 min followed by destaining with 1 M NaCl. The level of xylanase activity was recorded as unit area of degradation (circle diameter) of xylan. Statistical analysis was performed by calculating standard error of mean of each sample with two replications.

### In-gel chitinase assay

Crude protein extracts from IPTG induced and uninduced control *E. coli* cells was separated by 12% SDS-PAGE containing 0.01% glycol chitin. Sample loading buffer was same as that of SDS-PAGE but without β-mercaptoethanol. After electrophoresis, gel was immersed in a 0.1 M sodium acetate buffer (pH 5.0) containing 1% (v/v) deionized Triton X-100 and incubated for 30 min on shaker. The gel was then transferred to a fresh 0.1 M sodium acetate buffer (pH 5.0), and incubated at 37°C in thermostated chamber overnight for renaturation. After incubation, sodium acetate buffer was removed and activity staining was done with silver stain. Fixation was performed in a liquid solution containing 50% methanol, 12% acetic acid, and 0.0185% formaldehyde, all in v/v. Gels were then incubated on a shaker for 10 min in 40% (v/v) ethanol and for 10 min in 30% (v/v) ethanol, in continuous order. Pre-treatment, rinsing, and silver impregnation were performed using standard methods. Then the reaction was stopped using 5% acetic acid. After the development was stopped, gel was washed initially with 30% (v/v) methanol for 20 min and later with 10% (v/v) methanol for 20 min and then stored in 10% (v/v) methanol at 4°C before drying. Silver-stained gel was dried with a gel dryer (Bio-Rad Laboratories, Richmond, CA) and preserved at room temperature (Marek et al., 1995).

### Antifungal bioassay of defense response genes

Uniform sized sclerotium of *R. solani* was placed at the center of the Petri plate containing Potato Dextrose Agar (PDA) media and the plate was incubated at 28°C in dark. Using cork borer, uniformly sized wells were made at equidistance from the sclerotia of *R. solani*. Crude protein extract containing DR proteins at four different concentrations as well as one mock sample were loaded in the wells and the plate was incubated at 28°C in dark for 18 h. The growth of the *R. solani* mycelium was measured with a digital camera.

### Analysis for *R. solani* cell wall integrity

For this 50 μg of total crude protein extract along with the DR protein was placed separately on 1-day-old *R. solani* cultures grown on different glass slides and covered with thin layer of PDA medium. As a control a separate slide covered with thin mycelium of *R. solani* was inoculated with solubilization buffer. After 4 h these slides were examined for the degradation of *R. solani* mycelial cell wall under the light microscope.

## Results and discussion

### *In silico* characterization of qSBR11-1 associated genes

The genomic region associated with the qSBR11-1 QTL contains 11 tandem repeats. The FGENESH gene prediction software was used to analyze the repeats revealing that each of the repeats contained a gene with a continuous ORF without introns, indicating historical gene duplication events. Analysis of the encoded proteins revealed the presence in all of them of a glycosyl hydrolase family 18 (GH18) domain and show a high degree of homology with GH18_hevamine_Xip1_class III chitinases. The GH18 domain is present in the xylanase inhibitor Xip-I, and the class III plant chitinases such as hevamine, concanavalin B, and PPL2. The putative identity of the tandem genes is quite revealing as *R. solani* uses a number of secretory enzymes during the process of infection, such as cellulases, pectinases, and xylanases responsible for the degradation of rice cell wall components. In response, plants use enzymes such as xylanase inhibitors and chitinases as weapons against pathogenic intruders. Blast searches revealed the presence of ESTs for all the 11 genes confirming that they are expressed in rice.

### Analysis of promoter elements

*In silico* promoter analysis was performed using the PlantCARE database in order to identify putative promoter elements present within the 2 kb upstream regions of the 11 putative chitinase genes. Our analysis identified a number of *cis* sequences with homology to regulatory elements some of which, such as ABRE, ARE, CGTCA motif G-box, and W-box are present in the promoter of many defense response genes like maize catalase (CAT) which play an important protective role during osmotic and oxidative stresses (Polidoros and Scandalios, 1999; Guan et al., 2000; Supplementary Figure 1 and Supplementary Table 1). ABRE is an abscisic acid responsive element (Marcotte et al., 1989), the CGTCA motif confers methyl jasmonate responsiveness (Rouster et al., 1997), ARE has a role in anaerobic respiration, G-box is related to light responsiveness (Schulze-Lefert et al., 1989). A W-box, related to fungal elicitor responsiveness, was also found in the promoter of several tandem genes (Rushton and Somssich, 1998; Eulgem et al., 2000; Nischiuchi et al., 2004). Within the remaining elements identified in the 11 promoters there is a mix of abiotic and biotic stress responsive elements. There is ample evidence suggesting that many genes are able to induce tolerance during different stress conditions as they are multifunctional in nature (Xiong and Yang, 2003; Ramírez et al., 2009; Singh et al., 2013). Specific phytohormones like ABA, SA, JA and ethylene may play a major role in the activity of such genes. For example, the *BOTRYTIS SUSCEPTIBLE1* (*BOS1*) gene is activated by ABA and JA and induces resistance against osmotic stress as well as necrotrophic pathogens (Mengiste et al., 2003). Taheri and Tarighi (2010) demonstrated the involvement of JA signaling in basal and induced defense response against *R. solani.* Studies aimed at understanding the role of ABA in rice during pathogen infection have provided seemingly contradictory results. Some reports show a negative correlation between ABA levels and resistance to different pathogens (Anderson et al., 2004; Koga et al., 2004; Xu et al., 2013; De Vleesschauwer et al., 2014; Nafisi et al., 2014, 2015; Takatsuji and Jiang, 2014), while others have strongly identified a positive role for this hormone in plant defense activation (Ton and Mauch-Mani, 2004; Adie et al., 2007; Hernández-Blanco et al., 2007; De Vleesschauwer et al., 2014; Rejeb et al., 2014). According to previous studies, ABA and Me-JA pathways play major roles in the plant signaling during plant defense response. Meta analysis and transcriptomic studies of plants challenged with the necrotroph *Phythium irregulare* revealed the presence of the ABA responsive element (ABRE) in the promoters of many of the defense response genes and highlighted the importance of ABA as a defense regulator (Adie et al., 2007; Wasilewska et al., 2008). The abundance of ABRE and Me-JA elements revealed by the *in silico* promoter prediction in present study suggest an important role for ABA and JA in the increased expression of the 11 genes after *R. solani* infection (described below).

### Differential expression analysis

**Figure 1:**
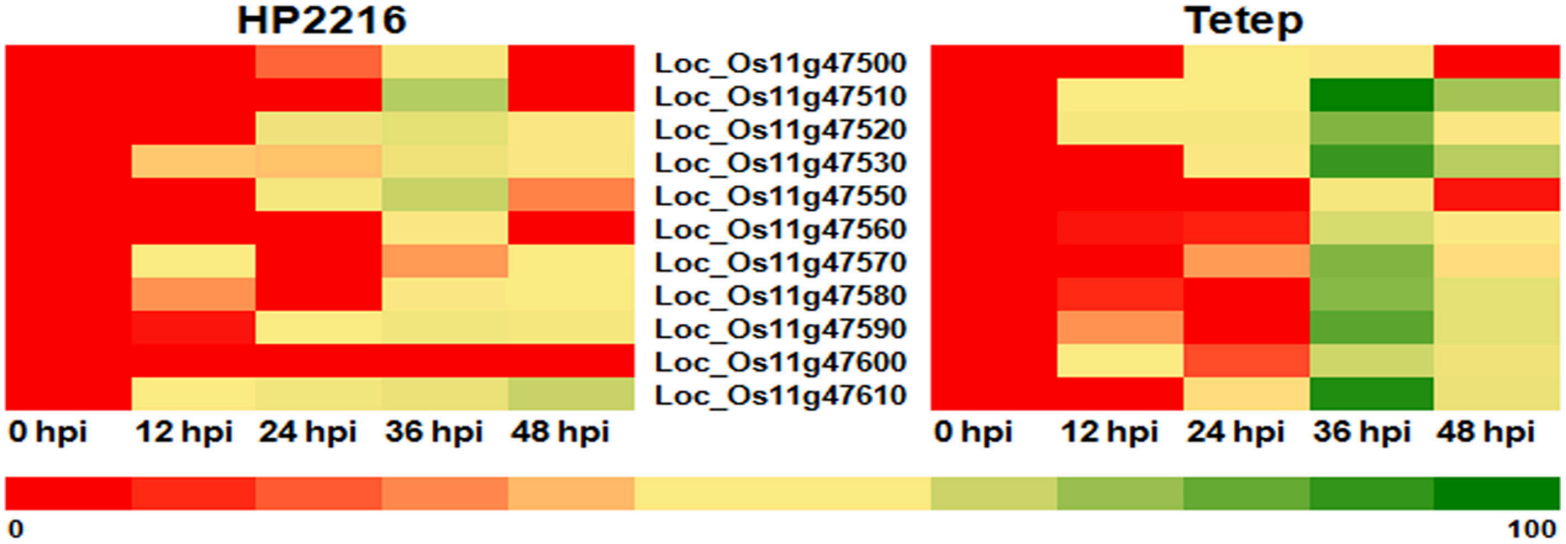
**Heat plot depicting the expression analysis in response to *R. solani* infection**. Rice lines HP2216 and Tetep were inoculated with *R. solani*, tissue samples were collected at different time points after inoculation and mRNA levels determined by qRT-PCR. While expression of most of the QTL qSBR11-1 associated genes is induced 36 h after infection, the levels of induction in the resistant Tetep line are much higher than in the susceptible HP2216 line. Color codes mentioned at the bottom of the figure show lowest (red color) and highest (green color) expression.

The expression pattern of the putative chitinase genes in response to *R. solani* infection was studied using quantitative real time PCR (qRT-PCR). Plants from the partially resistant Tetep and highly susceptible HP2216 rice lines were manually inoculated with the pathogen and tissue samples collected at 0, 12, 24, 36, and 48 h post infection (hpi). In the control HP2216 line, expression of most of the eleven tandem genes contained in the QTL region was upregulated 36 hpi with particularly strong induction of *LOC_Os11g47510* and *LOC_Os11g47610* (30 and 22 fold induction compared to non-inoculated controls; Figure 1). In the case of the partially resistant Tetep line, the level of induction in all but two of the eleven genes was much higher than those observed for the susceptible HP2216 line (Figure 1). Although, the temporal pattern of induction was similar in both lines, the induction levels were clearly different with *LOC_Os11g47510, LOC_Os11g47530, LOC_Os11g47590*, and *LOC_Os11g47610* showing 60–100 fold increase in expression; *LOC_Os11g47520, LOC_*Os11g47570, and LOC*_Os11g47580* induced in excess of 50 fold and *LOC_Os11g47560* and *LOC_Os11g47600* induced by ~20 fold (Figure 1). The changes in expression levels were well-correlated with the development and size of lesions on the rice sheath (Supplementary Figure 2). Lesion sizes at 0, 12, and 24 hpi were very small with a substantial increase observed at 36 hpi. The dramatic increase in lesion size coincided with the enhanced expression of the putative chitinase genes. At 48 hpi, water soaked lesions in Tetep changed into necrotic tissue while chitinase expression started to decrease. Our findings indicate that enhanced expression of the genes associated with the QTL qSBR11-1 could be responsible for the increased resistance shown by Tetep against *R. solani* infection. Recently Singh et al. (2012) have used Tetep as a donor of QTL qSBR11-1 and transferred it into indica rice line Improved Pusa Basmati 1 and the results indicate that qSBR11-1 imparts a significant level of field resistance against *R. solani* with the yield and quality on par with that of the recurrent parent. The 60–100 fold increase in gene expression levels observed in *LOC_Os11g47510, LOC_Os11g47530, LOC_Os11g47590*, and *LOC_Os11g47610* may be due to the presence of a comparatively higher number of *cis* elements like ABRE, ARE, CGTCA motif (Me-JA), G-box and W-box in their promoters while the genes showing lower levels of induction contain a lower number of elements.

The presence of numerous ABRE elements in the promoter of the 11 putative chitinase genes is very interesting. Experimental evidence suggests that ABA is a component of the signaling pathway activating plant defense against some, but not all the necrotrophic pathogens. Pre-treatment of rice plants with ABA before inoculation with the necrotrophic fungus *Cochliobolus miyabeanus* resulted in a drastic reduction of fungal spread in the mesophyll tissue compared to water-treated controls (De Vleesschauwer et al., 2010). On the other hand, Adie et al. (2007) found that ABA act as a positive regulator in the activation of the defense response against the necrotrophic fungus *Pythium irregulare* and as a negative signaling molecule in the case of other two necrotrophs, *Alternaria brassicola* and *Botrytis cinerea*. ABA enhances defense response at least through two independent mechanisms: callose priming and regulation of defense gene expression by mediating the activation of JA biosynthesis (Adie et al., 2007). The involvement of ABA and JA in plant defense adds significance to our finding that the promoters of the 11 QTL qSBR11-1 associated genes contain numerous ABA and JA related elements. A previous study involving rice—*R. solani* pathosystem had revealed that induction of chitinase genes starts 24 hpi in sheath blight resistant rice lines, while in susceptible lines chitinase induction was observed at 36 hpi (Shrestha et al., 2008). While our work also links chitinase expression with the development of the disease, in our case the differences observed between resistant and susceptible lines rely on the intensity of the response rather than the timing. Even though our data strongly suggest that activation of the chitinase genes plays an important role in the resistance against *R. solani* observed in Tetep, the exact mechanism for such resistance is still not properly understood.

### *In vitro* protein expression, xylanase inhibition, and chitinase activity assays

**Figure 2:**
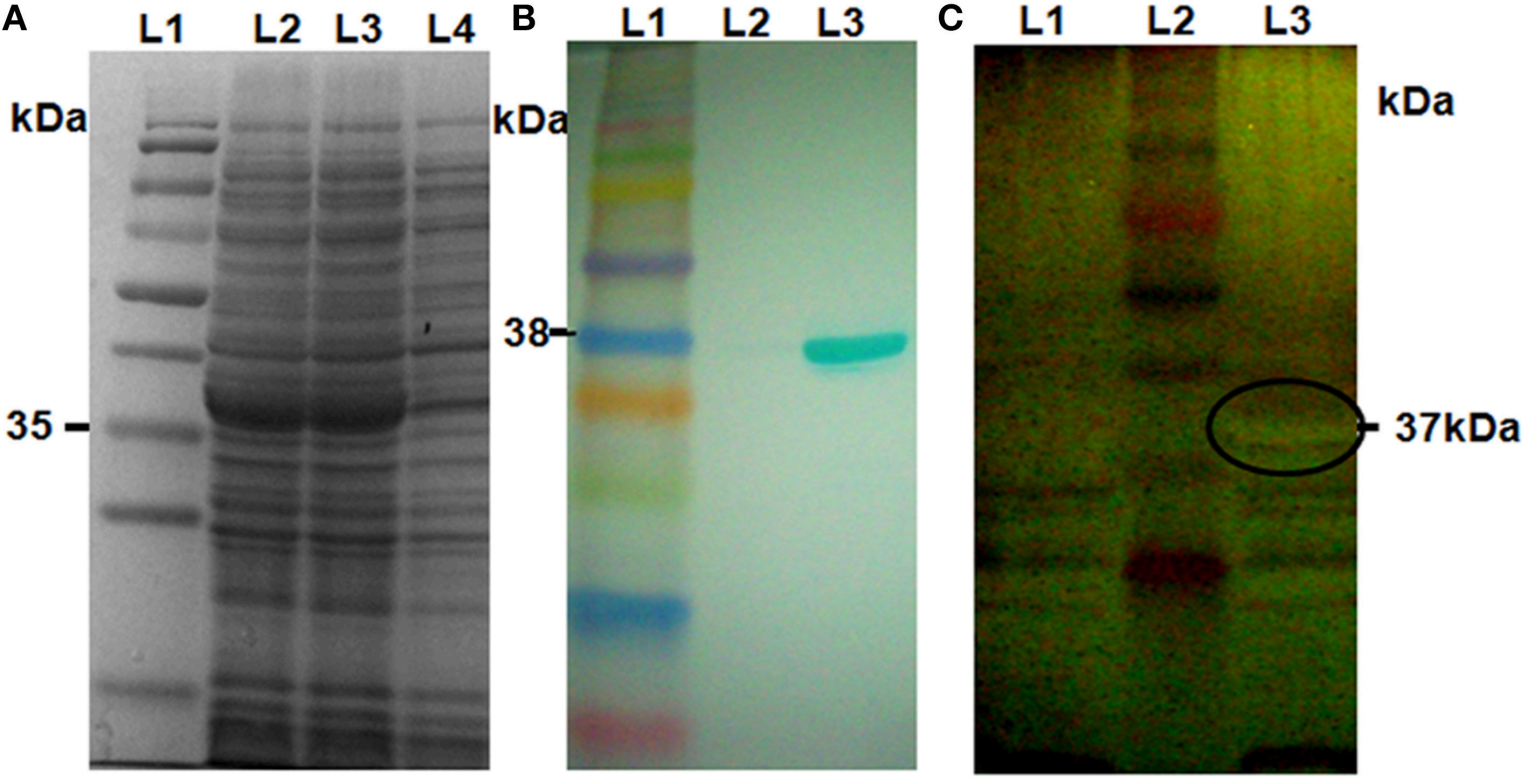
**Western blot and in-gel chitinase activity assay. (A)** Crude protein extracts from *E. coli* harboring an expression vector for *Loc_Os11g47510* (expected size of protein ~37 KDa) were separated by 12% SDS-PAGE, followed by coomassie blue staining. Lane 1, pre-stained protein ladder; Lanes 2 and 3, induced samples; Lane 4, non-induced sample. **(B)** Western blot using anti his-tag antibody; Lane1, Pre-stained protein ladder; Lane 2, non-induced sample; Lane 3, induced sample. **(C)** In-gel chitinase activity assay. Protein samples were separated on 12% SDS-PAGE gels containing 0.1% glycol chitin substrate and stained with silver stain. Lane 1, non-induced sample; Lane 2, pre-stained protein ladder; Lane 3, induced sample. The clear area of embedded glycol-chitin is marked in Lane 3.

We selected six putative chitinase genes for *in vitro* expression analysis based on the qRT-PCR expression data. Among them, three were highly expressed (Loc_Os11g47510, Loc_Os11g47530, Loc_Os11g47610); two moderately expressed (Loc_Os11g47520, Loc_Os11g47560), and one slightly expressed (Loc_Os11g47500). These six genes were cloned in the pET29a expression vector and protein production was induced in *E. coli* BL21. The SDS-PAGE analysis of the total crude protein extracted from the six recombinant pET29a clones showed that a protein of the expected size (35–40 kDa) was induced in all recombinant strains after induction with 0.1 mM IPTG (Figure 2A, Supplementary Figure 3A). The identity of the proteins was further confirmed by western-blot hybridization using anti his-tag antibodies (Figure 2B, Supplementary Figure 3B).

**Figure 3:**
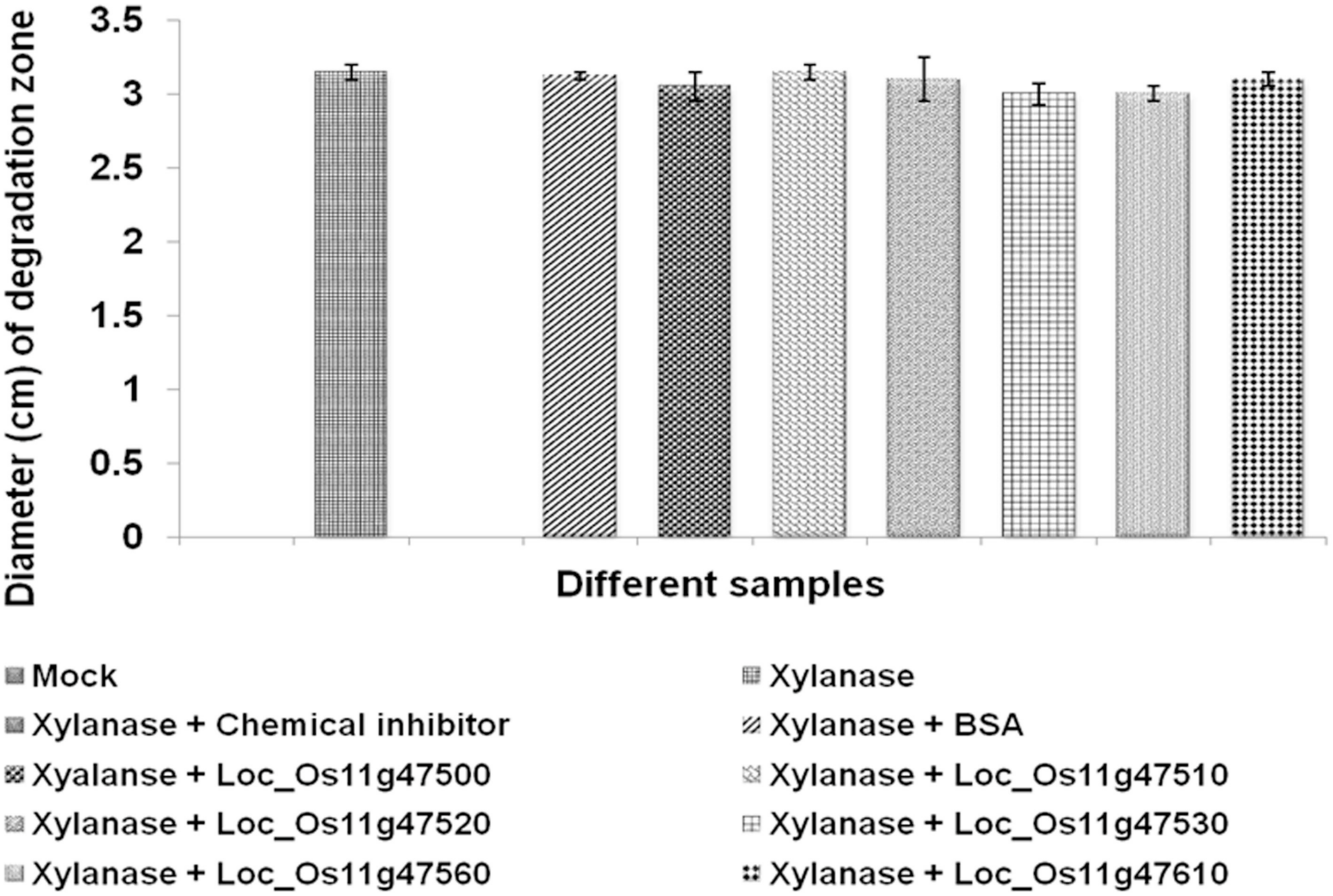
**Xylanase inhibition assay**. The possible inhibitory effect of *in vitro* produced proteins for six QTL qSBR11-1 associated genes on the activity of xylanase isolated from *T. langiginosus* was tested by performing xylanase activity assays on 1.5% LB agar plates containing 1% (w/v) birchwood xylan substrate followed by 1% Congo red stain. The level of xylanase activity was measured by measuring the diameter of degraded circles of xylan. Statistical analysis was performed by calculating standard error of mean of the two replicates for each of the protein.

Our initial bioinformatics analysis had revealed a high degree of homology between our candidate genes and the GH18_hevamine_Xip1_classIII chitinases, suggesting a possible function as either xylanase inhibitors or chitinases. In order to test the possible xylanase inhibitory activity we performed xylanase activity assays using standard fungal endo-1,4-β-xylanases isolated from *Thermomyces lanuginosus*. When endo-1,4-β-xylanases were incubated with crude extracts containing either of the six recombinant proteins, no inhibition in the activity of the xylanase was observed, whereas in the control experiment xylanase activity was inhibited after incubation with a synthetic xylanase inhibitor molecule (*N*-bromosuccinimide; Figure 3, Supplementary Figure 5). Previously, it was reported that xylanase activity is inhibited by XIP-I and TAXI-I class proteins, but due to the presence of salt bridges in wheat XIP-I protein, a xylanase inhibitor class protein did not show inhibitory activity (Payan et al., 2003). Similarly our results show that the six genes assayed in the present study do not possess xylanase inhibitor activity though they belong to class III XIPs.

To determine whether any of the proteins encoded by the six chosen genes had chitinase activity, we performed in-gel chitinase assays using the method described by Trudel and Asselin (1989). For this purpose we separated crude protein extracts from IPTG-induced and uninduced recombinant *E. coli* BL21 cultures on 12% SDS-PAGE gels containing Glycol chitin. All six proteins displayed in-gel chitinolytic activity was observed as a clear zone of embedded glycol-chitin of the expected size after the silver staining (Figure 2C, Supplementary Figure 4).

In previous studies, three different *XIPs, OsXIP*, rice *XIP*, and the putative rice xylanase inhibitor (*RIXI*) were found to be differentially expressed during various stress conditions. Among these three *XIPs*, bioinformatics analysis identified *OsXIP* as a class III chitinase. It was reported that *OsXIP* displays xylanase inhibitory activity but not chitinase activity (Durand et al., 2005; Goesaert et al., 2005; Takaaki and Esaka, 2007; Takaaki et al., 2008). Similar to the findings in the present study, *OsCLP*, an homolog of the TAXI-type xylanase inhibitor showed only chitinase activity despite of being a member xylanase inhibitor class of the proteins (Wu et al., 2013).

### Antifungal bioassay

**Figure 4:**
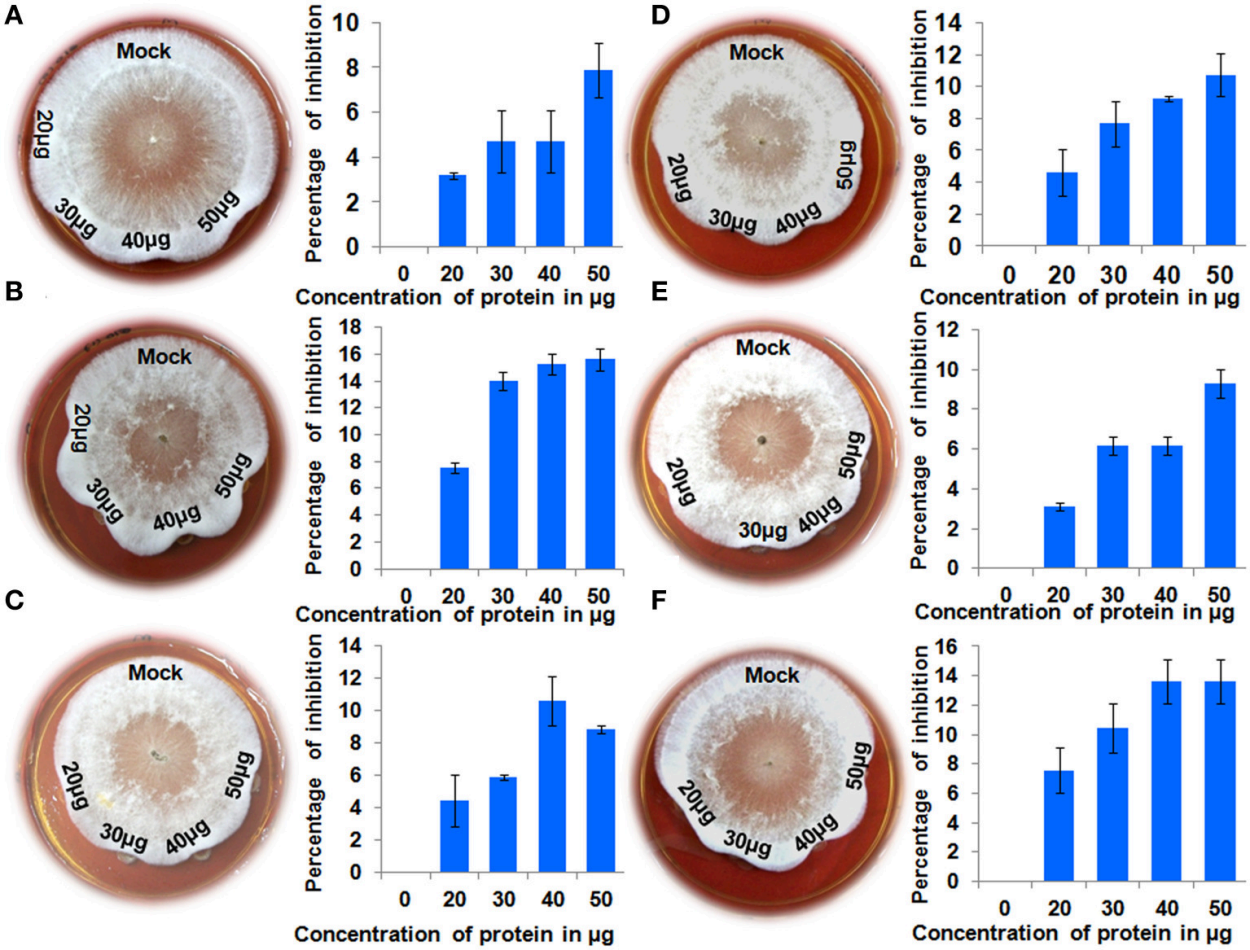
**Antifungal activity assays**. Different amounts (20, 30, 40, and 50 micrograms) of crude protein extract from induced *E. coli* samples harboring expression constructs for six QTL qSBR11-1 associated genes were placed into the wells created on PDA medium after 24 h of *R. solani* growth. A crude protein extract of *E. coli* carrying an empty pET29a was used as control. Percentage inhibition of *R. solani* growth was recorded after 24 h. Statistical analysis was performed by calculating standard error of mean of two replicates for each of the proteins. **(A-F)** Anti-fungal assays for genes *Loc_Os11g47500, Loc_Os11g47510, Loc_Os11g47520, Loc_Os11g47530, Loc_Os11g47560*, and *Loc_Os11g47610*, respectively.

**Figure 5:**
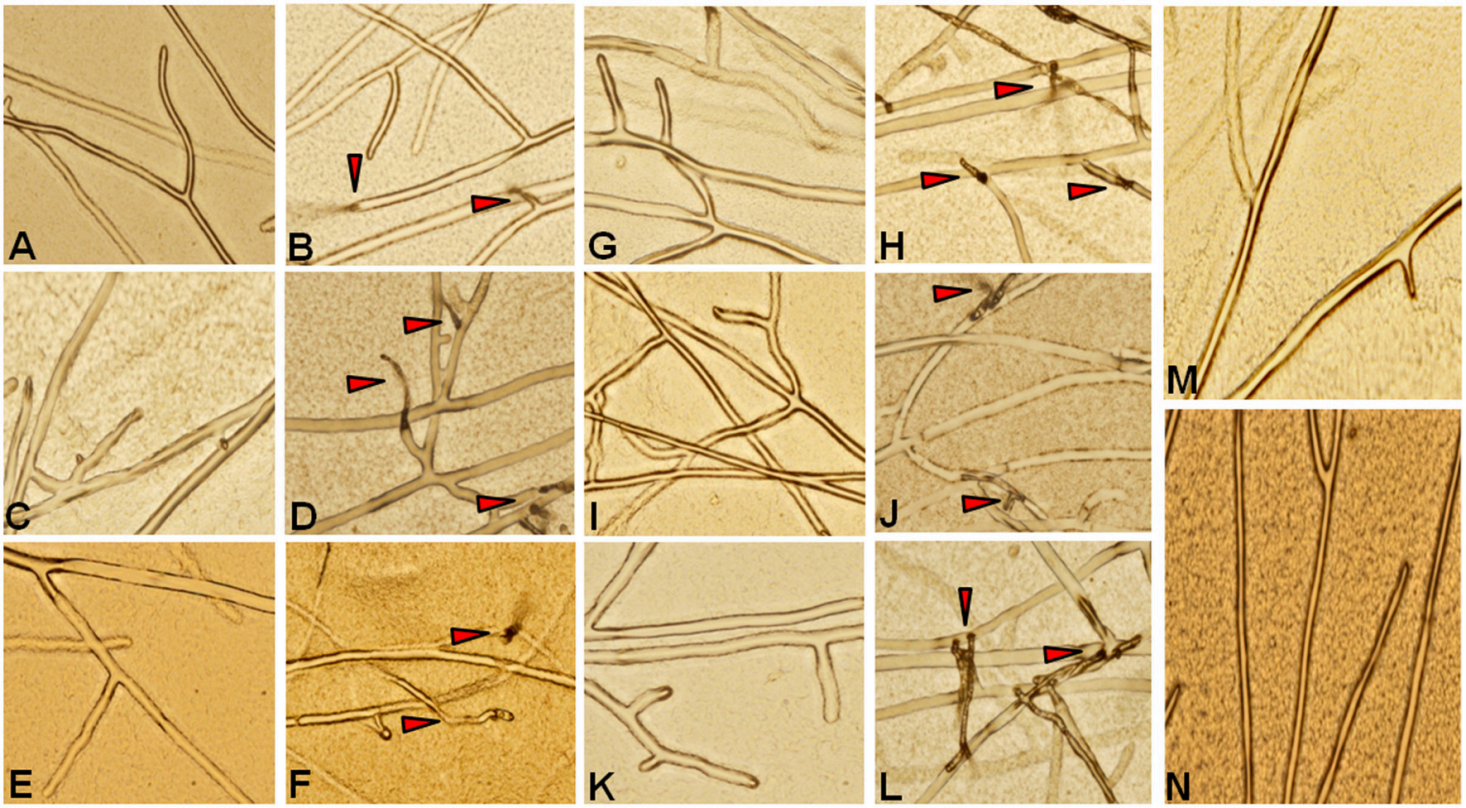
**Microscopic study of *R. solani* mycelia treated with recombinant proteins**. Crude protein extracts (50 μg) from induced *E. coli* samples harboring expression constructs for six QTL qSBR11-1 associated genes were added to 1-day-old *R. solani* cultures grown on a slide with a thin layer of PDA medium. The slide was examined for degradation of *R. solani* cell wall under a microscope. **(A,C,E,G,I,K);** *R. solani* in the presence of uninduced protein samples for *Loc_Os11g47500, Loc_Os11g47510, Loc_Os11g47520, Loc_Os11g47530, Loc_Os11g47560*, and *Loc_Os11g47610*, respectively. **(B,D,F,H,J,L);** *R. solani* mycelia in the presence of induced protein samples for *Loc_Os11g47500, Loc_Os11g47510, Loc_Os11g47520, Loc_Os11g47530, Loc_Os11g47560*, and *Loc_Os11g47610*, respectively. **(M)**, Uninduced pET29a and **(N)**, induced pET29a. Arrows indicate the degraded mycelium.

Recombinant proteins for all six genes were tested for their antifungal activity against the pathogen *R. solani*. Crude protein extracts at different concentrations were inoculated along with the pathogen and a mock sample (crude protein extract from an *E.* coli strain carrying an empty pET 29a) as control. Clear inhibition of *R. solani* hyphal growth was observed in the protein extracts of all the chitinases, whereas no inhibition was observed in the mock treated experiment (Figure 4). This experiment shows that all the proteins assayed display antifungal activity against the sheath blight pathogen *R. solani.* To analyze changes in the hyphal structure caused by the protein extracts, hyphae from all the six treatments were observed under the microscope. Microscopic observations revealed the degradation of hyphal structures in all the experiments except in the control (Figure 5). A previous study involving a chytinolytic bacteria *Enterobacter* sp. KB3 also showed that a chitinase protein extracted from this bacterium too degraded *R. solani* hyphae (Benhamou et al., 1993).

## Conclusion

**Figure 6:**
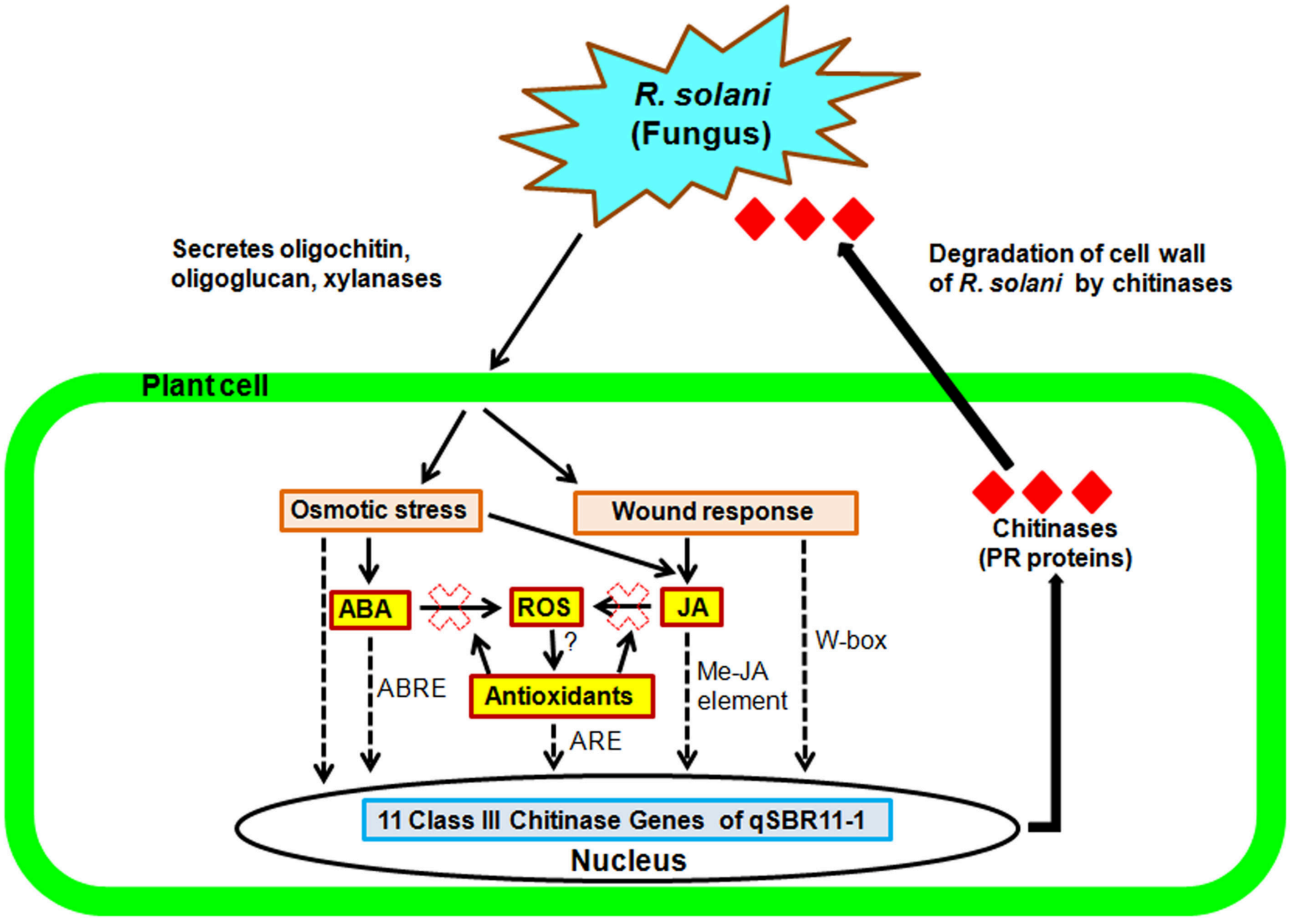
**Hypothetical model for the increased resistance conferred by the QTL qSBR11-1 associated genes during rice-*R. solani* interaction**. Pathogen infection triggers osmotic stress and wound responses mediated by ABA and JA signaling pathways activating the ABRE and Me-JA elements present in the promoters of the QTL qSBR11-1 associated genes. In addition, ABA and JA synergistically induce production of reactive oxygen species (ROS) and antioxidant molecules which will activate the ARE elements also present in the promoter regions. The combined and simultaneous effect of the ABA, MeJA, and ROS dependent elements results in the extremely strong induction observed for most of the chitinase genes. Solid thin lines represent the general pathway, dotted and solid bold lines represent our hypothetical model.

In the present study, expression analysis of 11 class III chitinase genes identified in the mapped QTL qSBR11-1 of rice line Tetep was performed and among them six genes were cloned and characterized. The expression of all 11 genes was inducible after the infection with *R. solani* in rice line Tetep suggesting that these genes are pathogen responsive (PR) in nature (Figure 6). Six of the eleven genes were further characterized and found to have chitinolytic activity but not xylanase inhibitor activity. Our study found a significant reduction in the mycelial growth of *R. solani* in the presence of the protein products of these chitinase genes. These genes will further enable the breeding process of rice for the management of sheath blight disease.

## Author contributions

TS: Conceived and designed the experiments; RK, IT, AK, and MK: Performed the experiments; RK, IT, HS, BD, and MK: Analyzed the data; TS, RK, BN, RN, VS, and JB: Wrote the paper.

### Conflict of interest statement

The authors declare that the research was conducted in the absence of any commercial or financial relationships that could be construed as a potential conflict of interest.
