# Supplementary material for: Functional Characterization of Novel Chitinase Genes Present in the Sheath Blight Resistance QTL: qSBR11-1 in Rice Line Tetep
Source: Front Plant Sci. 2016 Mar 1;7:244. doi: 10.3389/fpls.2016.00244 (PMC4771751; doi:10.3389/fpls.2016.00244)
Supplement: Supplementary file 1 [file Table1.DOCX]

**Supplementary Table 1.** Identification of promoter elements present in the upstream region of the 11 defense response genes.

| **^Promoter^**  **^elements^** | **^Loc_Os11g47500^** | **^Loc_Os11g47510^** | **^Loc_Os11g47520^** | **^Loc_Os11g47530^** | **^Loc_Os11g47550^** | **^Loc_Os11g47560^** | **^Loc_Os11g47570^** | **^Loc_Os11g47580^** | **^Loc_Os11g47590^** | **^Loc_Os11g47600^** | **^Loc_Os11g47610^** |
| --- | --- | --- | --- | --- | --- | --- | --- | --- | --- | --- | --- |
| ^ABRE^  ^Kayum et al. (2014)^ | ^37 (CACGTG)^ | ^-894, -15 (CACGTG)^  ^-646, -17 (CGTACGTCA)^ | ^-966, -87 (CACGTG)^ | ^-^ | ^-^ | ^65 (TACGTG)^ | ^-1395 (GCCGCGTGGC)^ | ^74 (CCTACGTGGC)^ | ^-993 (TACGTG),^  ^-686 (CACGTG)^ | ^+479 (GCCACGTACA)^ | ^-^ |
| ^ARE^  ^Kayum et al. (2014)^ | ^-^ | ^-998 (TGGTTT)^ | ^-^ | ^-289 (TGGTTT)^ | ^-901, -680, -513^  ^(TGGTT)^ | ^-1306, -559 (TGGTTT)^ | ^-1619, -1598, -1518^  ^-596, -375 (TGGTTT)^ | ^-^ | ^-180 (TGGTTT)^  ^119 (TGGTTT)^ | ^-^ | ^-496, +180 (TGGTTT)^ |
| ^G-Box^  ^Wang, et al. (2011)^ | ^37 (CACGTG)^ | ^-894, -15 (CACGTG)^  ^-896 (GCCACGTGGA)^ | ^-967, -88 (CACGTG),^  ^-86 (GCCACGTGGA)^ | ^-^ | ^-184 (CACGTA),^  ^78 (CACGTA)^ | ^-1027 (CACGAC),^  ^64 (GTACGTG)^ | ^-1101,^  ^(CACACATGGAA),^  ^-727 (CACGAC)^ | ^-1122 (CACGAC),^  ^-644 (CACGTT),^  ^130 (CACGTT)^ | ^-1061 (TCACACGTGGC),^  ^-993 (TACGTG),^  ^-686, -434 (CACGTG)^  ^319(CACGAC)^ | ^+481 (CACGTA),^  ^+225 (CACGTT)^ | ^+163 (CACGTA), +253 (CACGAC)^ |
| ^CGTCA-motif^  ^Kayum et al. (2014)^ | ^-^ | ^-^ | ^-^ | ^-1735, -89 (CGTCA)^ | ^-1234 (CGTCA)^ | ^-^ | ^-^ | ^10 (CGTC)^ | ^-1005, -593 (CGTCA)^ | ^-95, +137 (CGTCA)^ | ^-^ |
| ^W-Box^  ^Kayum et al. (2014)^ | ^-^ | ^-^ | ^-1039 (TTGACC)^ | ^-938, -573 (TTGACC)^ | ^-^ | ^-^ | ^-^ | ^-607 (TTGACC)^ | ^-^ | ^-^ | ^-^ |
| ^5´ UTR Pyr-rich stretch^  ^Kayum et al. (2014)^ | ^-^ | ^-^ | ^-^ | ^-^ | ^-^ | ^-^ | ^-^ | ^-^ | ^-^ | ^-^ | ^+318 (TTTCTTCTCT)^ |
