# Supplementary material for: Functional Characterization of Novel Chitinase Genes Present in the Sheath Blight Resistance QTL: qSBR11-1 in Rice Line Tetep
Source: Front Plant Sci. 2016 Mar 1;7:244. doi: 10.3389/fpls.2016.00244 (PMC4771751; doi:10.3389/fpls.2016.00244)
Supplement: Supplementary file 2 [file Table2.DOCX]

**Supplementary Table 2**. List of qRT-PCR primers used for expression analysis study of 11 defense response genes in response to *R. solani*

| **Gene Id** | **Primer’s Sequence (5’-3’)** | **Tm** | **Amplicon Length** |
| --- | --- | --- | --- |
| LOC_Os11g47500.F | CGAAAACCTCTACTACTCCGTCCTC | 56.27 | 164 |
| LOC_Os11g47500.R | GGATGTTCTGGTTGTAGTCGTTGAT | 55.15 |  |
| LOC_Os11g47510.F | CTCCTCTCCTTGTTAGCGGTGTTCTT | 58.32 | 163 |
| LOC_Os11g47510.R | GGAAGGAGATGATGACGATGTTGTA | 54.51 |  |
| LOC_Os11g47520.F | CGACGGCTACAACAAGTACTACC | 55.22 | 275 |
| LOC_Os11g47520.R | AGTAGAGATCCTTCTGAAACATCCA | 52.97 |  |
| LOC_Os11g47530.F | AAGCACTGCCAGTCCAAGTACATC | 57.17 | 260 |
| LOC_Os11g47530.R | ATCTCGAACTTGTAGTGGCTGAACA | 56.26 |  |
| LOC_Os11g47550.F | AGGAAGCTCTACAGCTACAGAAACA | 55.47 | 298 |
| LOC_Os11g47550.R | TGTACATCACCAGCGTGTAGTAGAG | 55.96 |  |
| LOC_Os11g47560.F | TTCTACAACGTCTTCGGCTACCAG | 56.51 | 207 |
| LOC_Os11g47560.R | GAGGTAGGCGTTCCAGAGGTTATC | 56.36 |  |
| LOC_Os11g47570.F | GGTACATGTTCCAAAAGGACCTCTA | 61.72 | 122 |
| LOC_Os11g47570.R | CTCACCACTGTAATTGGCCTTCTT | 62.82 |  |
| LOC_Os11g47580.F | CAAGAACATCTTCGTCTTCCTCTC | 53.32 | 244 |
| LOC_Os11g47580.R | GTACATCTTGTTGTAGGCGTAGAGGT | 56.31 |  |
| LOC_Os11g47590.F | GTTCTACGACGACAGGAGGTGCTC | 58.86 | 273 |
| LOC_Os11g47590.R | ATGCCTTTGATGGTCTGACCGTAG | 56.76 |  |
| LOC_Os11g47600.F | ATAAACCCGGAGTCGCTCTACTA | 54.47 | 116 |
| LOC_Os11g47600.R | CCGTAGTAGTTATCGTAGGCTTTGT | 54.33 |  |
| LOC_Os11g47610.F | AGAGCTCGTGCGACTACAACTACT | 57.49 | 229 |
| LOC_Os11g47610.R | GTGATCTTGTCGTAGTAGCGATCC | 55.06 |  |
| 18S_rRNA. F | CTACGTCCCTGCCCTTTGTACA | 63.0 | 65 |
| 18S_rRNA. R | ACACTTCACCGGACCATTCAA | 62.0 |  |
