# Supplementary material for: Functional Characterization of Novel Chitinase Genes Present in the Sheath Blight Resistance QTL: qSBR11-1 in Rice Line Tetep
Source: Front Plant Sci. 2016 Mar 1;7:244. doi: 10.3389/fpls.2016.00244 (PMC4771751; doi:10.3389/fpls.2016.00244)
Supplement: Supplementary file 3 [file Table3.DOCX]

**Supplementary Table 3.** List of gene specific primers used for cloning and expression analysis with restriction sites *EcoRI* (GAATTC) and *HindIII* (AAGCTT)

| **Gene Id** | **Primer’s Sequence (5’-3’)** | **Tm** |
| --- | --- | --- |
| LOC_Os11g47500.F | GTATCCGAATTCATGGCGTCCCAACGCCGGCGATCATCT | 56.27 |
| LOC_Os11g47500.R | CTATCGCAAGCTTAGCCAAGTCCTTGACGTATCGGCCGTAGTG | 55.15 |
| LOC_Os11g47510.F | GTATCCGAATTCATGGCGTCCCAACGCCGGCGATCATCC | 58.32 |
| LOC_Os11g47510.R | GATGCCAAGCTTAGCCCAGTGCTTGACATATCTTCCGTAGTG | 54.51 |
| LOC_Os11g47520.F | GTATCCGAATTCATGGGCCTCGTGCACGCACTCCT | 55.22 |
| LOC_Os11g47520.R | GTATGCAAGCTTACCCTCACCAGTGTAGTTGGCCTTCTTG | 52.97 |
| LOC_Os11g47530.F | GTATCCGAATTCATGGCGTTCGGACGCCGTTC | 57.17 |
| LOC_Os11g47530.R | GGTTGCAAGCTTAAAAACTTTTCCAGCGGAGTAG | 56.26 |
| LOC_Os11g47560.F | TGATCCGAATTCATGAAGATGAAGGCTCTCCTCC | 56.51 |
| LOC_Os11g47560.R | GATACGAAGCTTGCCCTCACCACTGTAGTTG | 56.36 |
| LOC_Os11g47610.F | TGATCCGAATTCATGTCGAAGCTCCAACTCCG | 57.49 |
| LOC_Os11g47610.R | GATCCGAAGCTTGACAATGTCTTTGATGGCACG | 55.06 |
