# Supplementary figures and images for: Functional Characterization of Novel Chitinase Genes Present in the Sheath Blight Resistance QTL: qSBR11-1 in Rice Line Tetep
Source: Front Plant Sci. 2016 Mar 1;7:244. doi: 10.3389/fpls.2016.00244 (PMC4771751; doi:10.3389/fpls.2016.00244)

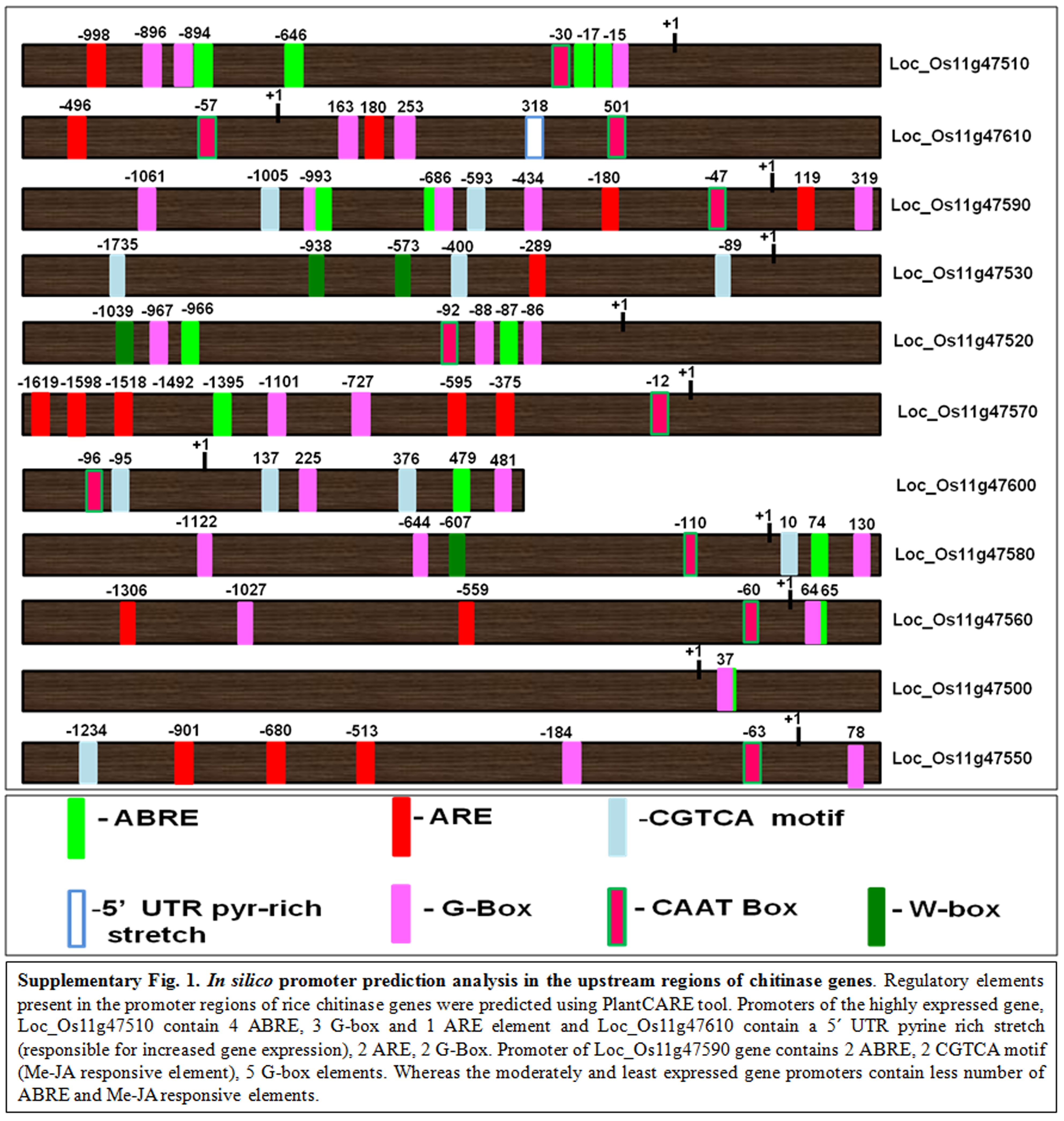

Supplement: Supplementary file 4 [file Image1.TIF]

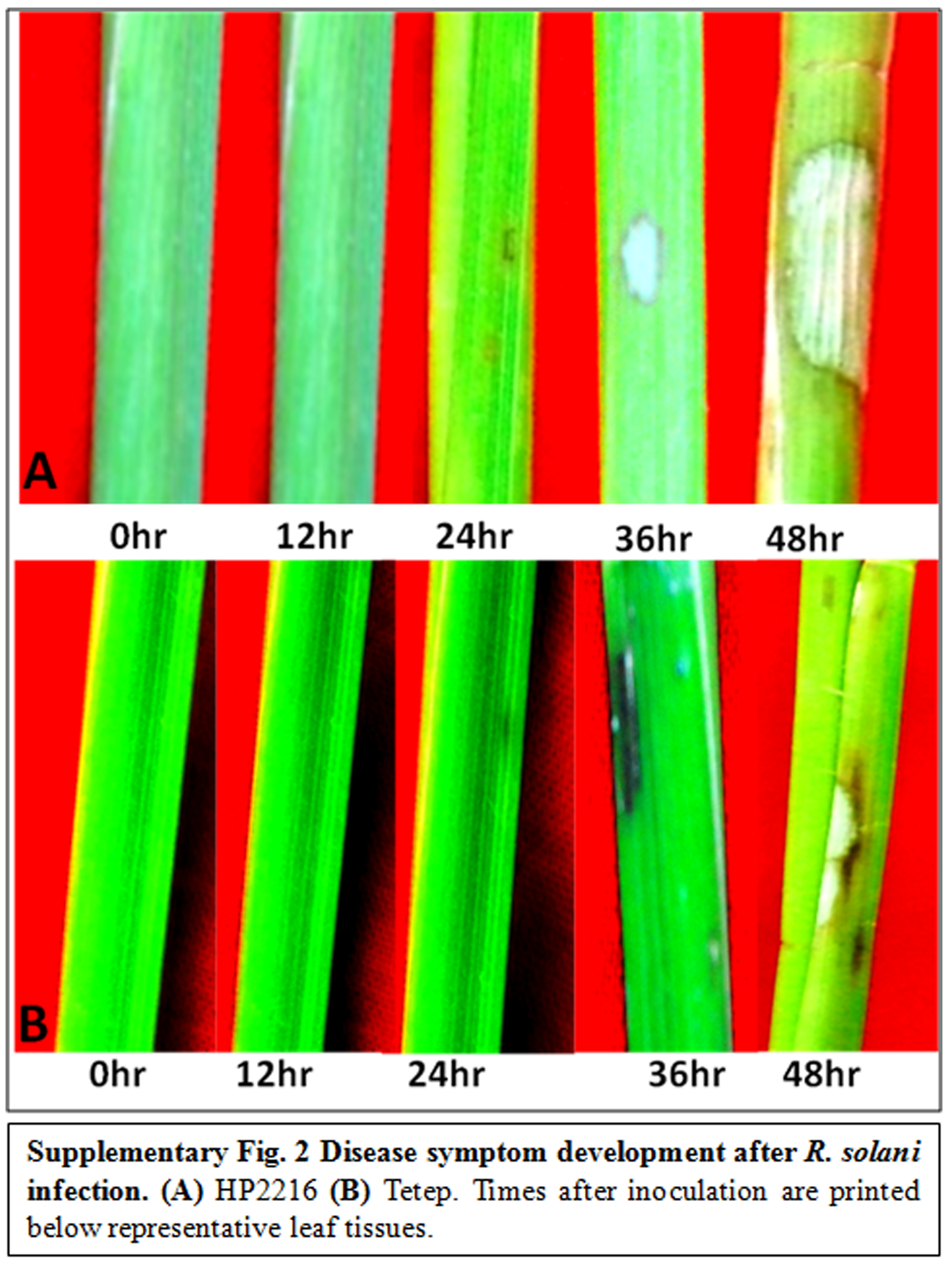

Supplement: Supplementary file 5 [file Image2.TIF]

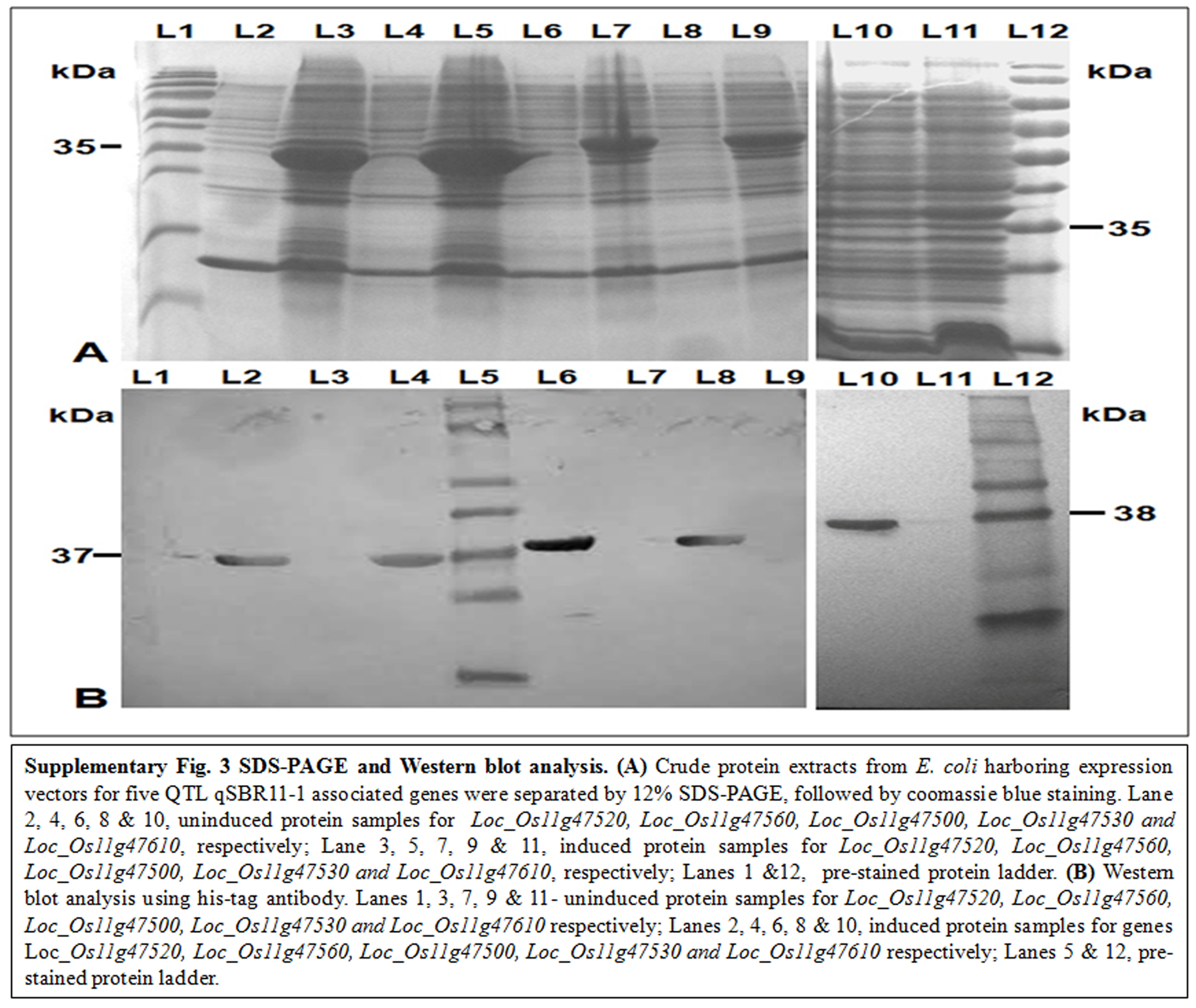

Supplement: Supplementary file 6 [file Image3.TIF]

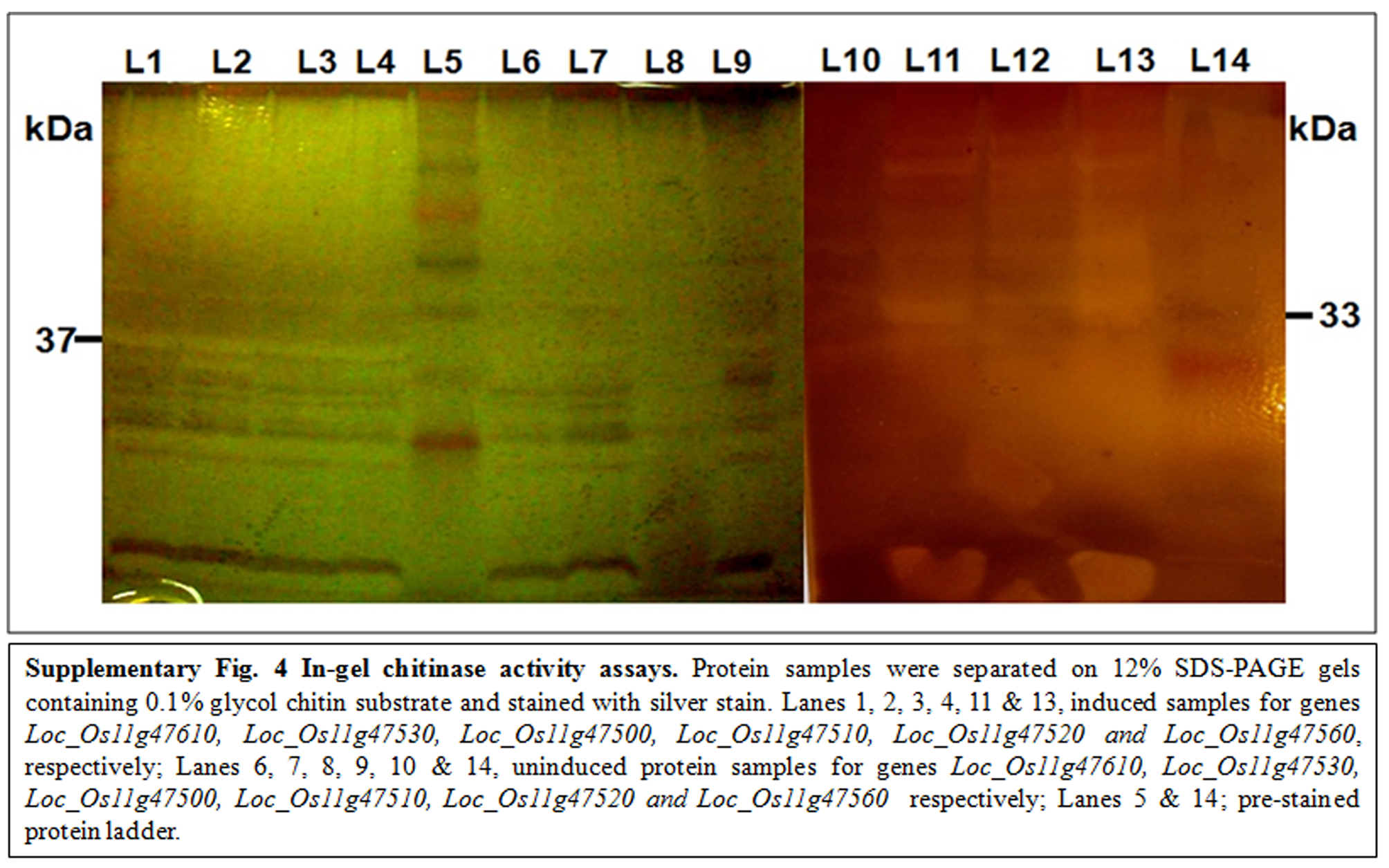

Supplement: Supplementary file 7 [file Image4.TIF]

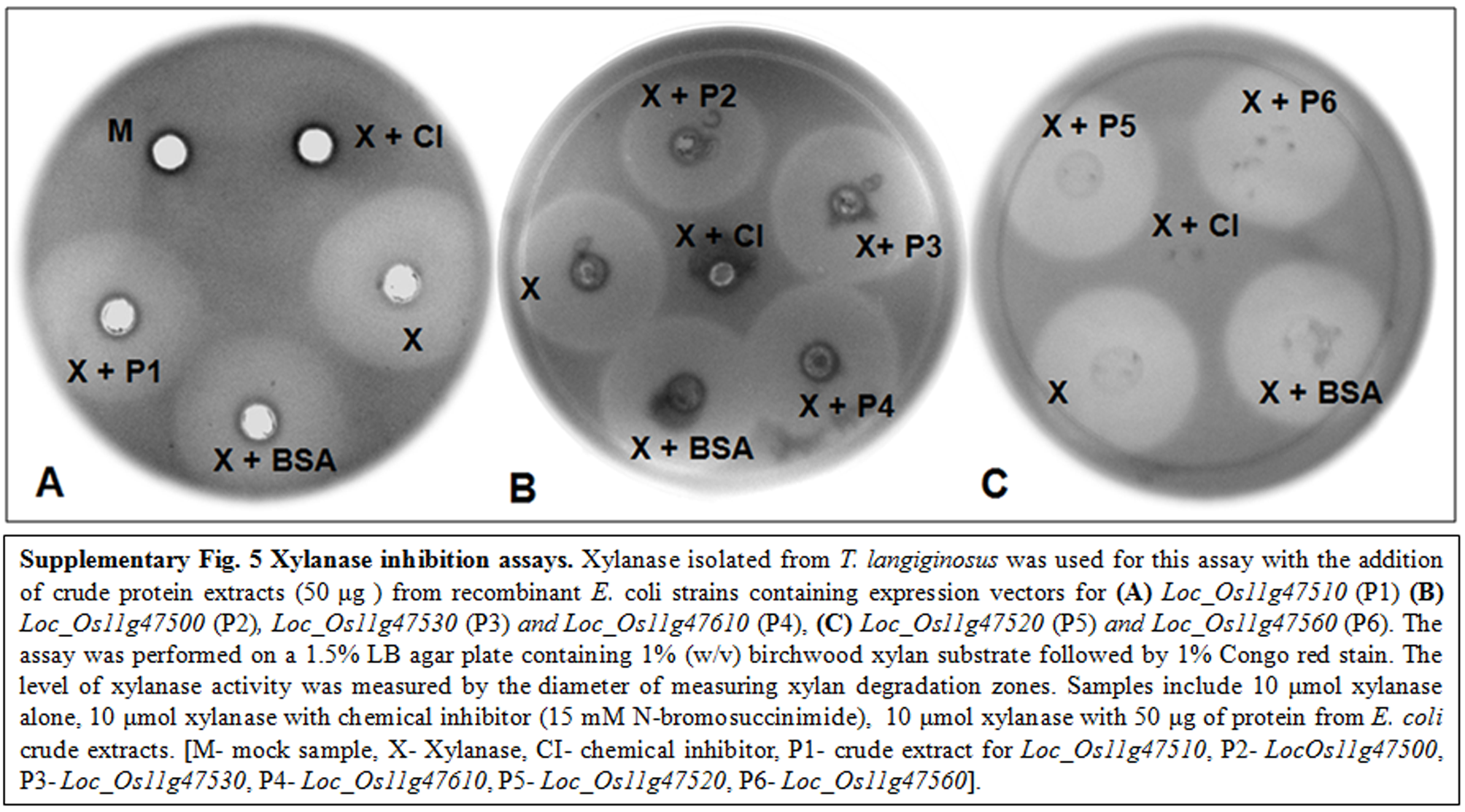

Supplement: Supplementary file 8 [file Image5.TIF]
